# Non-High-Risk Neuroblastoma: Classification and Achievements in Therapy

**DOI:** 10.3390/children6010005

**Published:** 2019-01-08

**Authors:** Holly J. Meany

**Affiliations:** Center for Cancer and Blood Disorders, Children’s National Health System, The George Washington University School of Medicine and Health Sciences, Washington, DC 20010, USA; hmeany@childrensnational.org; Tel.: +1-202-476-2800

**Keywords:** neuroblastoma, intermediate risk neuroblastoma, classification, treatment, low-risk neuroblastoma

## Abstract

Neuroblastoma, a tumor of the sympathetic nervous system, is the most common extra-cranial neoplasm of childhood. Variables with prognostic significance in patients with neuroblastoma, including age at diagnosis, disease stage, tumor histology, *MYCN* gene amplification, tumor cell ploidy, and the presence of segmental chromosomal aberrations are utilized to classify patients based on risk of disease recurrence. Patients with non-high-risk neuroblastoma, low- and intermediate-risk categories, represent nearly half of all newly diagnosed cases. This group has an excellent event-free and overall survival with current therapy. Over time, the objective in treatment of non-high-risk neuroblastoma has been reduction of therapy intensity to minimize short- and long-term adverse events all the while maintaining excellent outcomes.

## 1. Introduction

Neuroblastoma, a tumor of the sympathetic nervous system, is the most common extra-cranial neoplasm of childhood accounting for approximately 8–10% of childhood cancers. The incidence is approximately 10 cases per 1 million per year in children under 15 years of age in the United States. Neuroblastoma is a disease of young children; more than one third of patients are diagnosed during infancy, and approximately 90% are diagnosed before five years of age [1].

Neuroblastoma tumors most commonly arise in the adrenal medulla or along the sympathetic chain. A majority of patients have abdominal disease resulting in symptoms of pain, distention, constipation or bowel, and/or bladder dysfunction. Extra-abdominal disease may occur in the paravertebral ganglia of the neck, chest or pelvis and result in a palpable mass, respiratory symptoms, neurologic compromise or spinal cord impingement if tumors invade the neural foramina. Horner syndrome is a common manifestation of superior cervical ganglion involvement.

More than half of patients with neuroblastoma have metastatic disease at the time of diagnosis. Typical metastatic sites include regional or distant lymph nodes, cortical bone, bone marrow, and liver while involvement of the lungs and brain is rare. Metastatic disease may result in constitutional symptoms such as fever, weight loss or fatigue as well as pancytopenia, periorbital ecchymosis, and bone pain at the time of diagnosis.

Current risk classification utilizes clinical factors at diagnosis (age, disease stage) and tumor biologic features to assign patients to appropriate therapy based on risk of disease recurrence. Non-high-risk neuroblastoma, low- and intermediate-risk categories, represent nearly half of all newly diagnosed patients.

## 2. Classifying Patients with Non-High-Risk Neuroblastoma

Variables with prognostic significance in patients with neuroblastoma, including age at diagnosis, disease stage, tumor histology, *MYCN* gene amplification, tumor cell ploidy, and the presence of segmental chromosomal aberrations are utilized to classify patients and assign appropriate treatment regimen.

### 2.1. Age at Diagnosis

Prior studies have demonstrated patients less than one year of age at diagnosis have a more favorable outcome as compared to older patients. Despite a higher proportion of younger patients presenting with localized disease, age remained a significant prognostic factor even after stage was taken into consideration [2]. In the more recent era, international cooperative group analyses have provided evidence to support an increase to 18 months of age at diagnosis as an appropriate cutoff for separating younger from older patients with respect to risk stratification [3,4].

### 2.2. Disease Staging

The presurgical International Neuroblastoma Risk Group Staging System (INRGSS) [5,6], based on radiographic characteristics of the primary tumor and presence or absence of metastatic disease, has replaced the International Neuroblastoma Staging System (INSS) [7], a post-surgical staging system. The INRGSS distinguishes locoregional tumors based on the absence (stage L1) or presence (stage L2) of image-defined risk factors, a measure of tumor invasiveness. Patients with distant metastatic disease are classified as stage M disease, and those infants with the characteristic metastatic pattern of liver, skin, and limited bone marrow involvement are classified as stage MS disease (Table 1).

### 2.3. Tumor Histology

Neuroblastoma arises from primitive sympathetic nerve cells derived from the neural crest. A spectrum can be observed histologically including benign ganglioneuroma composed of mature, fully differentiated cells, ganglioneuroblastoma containing both differentiated ganglion cells and malignant neuroblastoma cells and the classic malignant neuroblastoma tumors. The International Neuroblastoma Pathology Classification System (INPC) relates histopathologic features to clinical outcome and is currently used for risk classification. Tumors are categorized as favorable or unfavorable histologic subtype based upon the degree of neuroblast differentiation, Schwannian stroma content, mitosis-karyorrhexis index (MKI), and age at diagnosis [8].

### 2.4. Tumor Genomic Features

Distinct tumor genomic features are also critical in characterizing patients with neuroblastoma. Amplification of the *MYCN* gene on chromosome 2 is the most common genomic alteration in neuroblastoma, occurring in approximately 20% of patients, and is highly correlated with advanced disease stage and a poor prognosis. Due to the poor outcomes observed in patients with *MYCN* amplification, these children are currently treated with intensive multi-modality (high-risk) therapy [9,10,11,12].

Tumor cell ploidy (DNA index) is a strong prognostic marker, particularly for younger patients with metastatic disease. Hyperdiploid DNA content correlates with favorable disease behavior, more commonly found in lower stage disease and in *MYCN* non-amplified tumors. Near-diploid (and near-tetraploid) tumors tend to behave more aggressively. This correlation is highly predictive in patients less than one year of age, but is no longer significant after 18 to 24 months of age [13,14].

Retrospective studies have shown loss of heterozygosity (LOH) of 1p or 11q predict poor outcome for subsets of neuroblastoma patients. The DNA index and 1p and 11q LOH have more recently been used in North American and European clinical trials as integral biomarkers to determine risk groups and assign therapy [15,16,17,18,19,20,21]. More recently, whole-chromosome copy number profiles have been shown to be prognostic in neuroblastoma. Two overall genomic patterns have been described; genomes characterized by numerical or whole-chromosome gains and losses, termed numerical chromosomal aberrations (NCAs), and genomes characterized by segmental gains and losses of regions within chromosomes, segmental chromosomal aberrations (SCAs). Numerical chromosomal aberrations are associated with more favorable outcomes while SCAs portend a poorer prognosis and are indicative of higher risk disease. Even within the cohort of patients classified as non-high-risk neuroblastoma, tumors found to have SCAs have an inferior progression-free and event free survival (EFS) [15,22,23,24,25].

There are additional genomic features that may be useful particularly in the non-high-risk neuroblastoma group to distinguish patients with a more favorable outcome from those who do not fare as well. Select examples include Ha-*ras* p21 gene expression [26], positive *TrkA* and low affinity nerve growth factor receptor mRNA expression [27,28], parental imprinted miRNA (maternal miR-487b and paternal miR-516a-5p) expression [29]. A genome-wide association study identified four genes (*DUSP12*, *DDX4*, *IL31RA* and *HSD17B12*) associated with low risk disease [30]. Gene expression profiling has also been demonstrated by several groups to be a reliable method of classifying patients with non-high-risk neuroblastoma [31,32,33]. Though not a part of the current classification system these factors may impact future schema.

### 2.5. Classification Schema

Incorporating these known prognostic features, the Children’s Oncology Group (COG) (Table 2) [34] and INRG (Table 3) [5] have developed classification schema. In the INRG schema, non-high-risk neuroblastoma patients include those with localized disease, all L1 tumors and L2 tumors without *MYCN* gene amplification. Also included are patients with metastatic disease, <18 months of age at diagnosis with *MYCN* non-amplified tumors and those with stage MS disease, *MYCN* non-amplified tumors except patients with an unfavorable biologic feature (11 q aberration).

## 3. Non-High-Risk Neuroblastoma Treatment over Time

Therapy intensity in neuroblastoma varies significantly for patients in different risk groups (Table 4). Patients with low risk disease may be observed or undergo surgical resection. Intermediate risk treatment typically includes systemic chemotherapy with carboplatin, etoposide, cyclophosphamide, and doxorubicin administered at three-week intervals for up to eight cycles. Other active agents, topotecan for example, may be included in patients with refractory or relapsed disease and biologic therapy with isotretinoin is utilized in select cohorts with metastatic disease. Patients undergo surgical resection of the primary tumor in select cases. Radiation therapy is reserved for rare emergent situations. This compares with high-risk patients who are treated with multimodality intensive therapy [35,36].

Patients classified as having non-high-risk neuroblastoma (low- and intermediate-risk categories) have an excellent EFS and overall survival (OS) with current therapy, nearing 90% and exceeding 95% respectively in cooperative group trials [37,38]. Over time, the objective in treatment of non-high-risk neuroblastoma has been reduction of therapy intensity to minimize short- and long-term adverse events all the while maintaining excellent outcomes. As a result, patients are currently receiving fewer cycles of chemotherapy, surgical interventions are less aggressive and cohorts of the most favorable patients undergo observation only. This de-escalation of therapy was achieved through several consortium group clinical research trials described below.

### 3.1. Low-Risk Neuroblastoma

Patients with low-risk neuroblastoma (stage 1, 2, and rare 4S) treated on the COG P9641 trial, underwent maximally safe tumor resection followed by 2 to 4 cycles of chemotherapy for those with organ compromise, life threatening symptoms or less than a partial resection. The five-year EFS and OS for eligible patients (*n* = 915) was excellent, 89% and 97%, respectively. Importantly the EFS and OS rates for patients treated with surgery alone and for patients treated with surgery and chemotherapy were not significantly different nor were the rates of progressive disease or disease recurrence. Among patients with stage 2b disease, EFS and OS were significantly lower for those with unfavorable histology or diploid tumors, and OS was significantly lower for those over 18 months of age at diagnosis [37]. Similar results were seen on an International Society of Pediatric Oncology European Neuroblastoma (SIOPEN) study in patients with *MYCN* non-amplified localized neuroblastoma (LNESG1) [39].

### 3.2. Intermediate-Risk Neuroblastoma

Intermediate-risk patients on a Children’s Cancer Group trial (CCG 3881) with Evans stage III tumors classified as more favorable based on age, *MYCN* gene copy number, histology, and serum ferritin level were treated with less intensive therapy. This cohort included patients less than one year of age (*n* = 89) and patients one year of age or greater with favorable biology (*n* = 54). The four-year EFS and OS rates both were 100% for patients with favorable biology regardless of age and 90% and 93%, respectively, for patients less than one year of age with at least one unfavorable characteristic. Importantly for this group EFS did not depend on degree of resection, gross versus partial resection [40].

Patients with intermediate-risk, *MYCN* non-amplified neuroblastoma including infants (<365 days of age) with INSS stage 3 or 4 disease, children (≥365 days of age) with stage 3 disease with favorable histology, and infants who had stage 4S disease with unfavorable biologic features were also evaluated in the context of decreased therapy intensity (COG A3961). Patients who had disease with favorable biologic features (favorable histology and hyperdiploid tumors) received four cycles of chemotherapy and patients with unfavorable biologic features or an incomplete response received eight chemotherapy cycles as well as surgical resection in select cases. The three-year EFS and OS for the entire group (*n* = 479) were 88% and 96%, respectively, with a 98% OS for patients with tumors exhibiting favorable biologic features as compared to 93% for those with unfavorable biologic features. Ploidy was significantly predictive of outcome. Excellent outcomes were achieved for all disease stages, but EFS and OS were significantly higher for patients with stage 3 and 4S disease as compared to patients with stage 4 disease [38]. A SIOPEN study demonstrated comparable favorable outcomes in patients <18 months of age and those with favorable histology following reduction of therapy [41].

On a German trial, infants with *MYCN* non-amplified, localized neuroblastoma underwent either surgical resection or were observed without intervention. Chemotherapy was administered initially only to patients with life threatening symptoms. OS at three years was excellent for all cohorts; 99% for those observed without intervention (*n* = 93), 95% for those treated with chemotherapy (*n* = 57), and 98% for those who underwent surgical resection (*n* = 190). In the observation cohort, with a median follow up 58 months (range 10–128 months), spontaneous regression was demonstrated in 47% of patients. The age at first evidence of regression varied (median 6.7 months, range 1–23 months) and regression was complete in 17 patients and incomplete in 27 patients. Ten patients (11%) had no change in tumor size with observation, eight underwent complete surgical resection, one received chemotherapy and one continued observation only. Tumor progression was observed in 42%, but only 4.3% progressed to Stage 4 disease. A high percentage (70%) of patients were spared cytotoxic therapy and the associated acute and late effects [42].

These trials confirmed excellent outcomes can be achieved despite reduction in therapy for select patients and culminated in a recent phase III COG trial utilizing tumor biologic features to determine duration of treatment in patients with intermediate risk neuroblastoma (ANBL0531). Risk-stratification and treatment assignment was based on age at diagnosis, INSS stage, histology, *MYCN* status, DNA ploidy and loss of heterozygosity (LOH) at 1p36 and/or 11q23. Patients received 2–8 courses of chemotherapy with or without surgery and the treatment endpoint for patients with localized disease was partial response, greater than 50% decrease in size of the primary tumor, aimed to further reduce therapy. The three-year EFS and OS for all patients were 83% and 95%, respectively, supporting further decrease in therapy for many patients with intermediate risk neuroblastoma. It has been demonstrated that a partial response is sufficient to stop therapy in a majority of cases. Patients with unfavorable biology fared worse and are likely the exception to therapy reduction [43].

### 3.3. Localized Perinatal Tumors

Patients in the perinatal group typically have localized tumors that arise in the adrenal gland. Additionally, the vast majority of the perinatal tumors exhibit favorable biologic features (<5% *MYCN* amplified, >95% favorable histology), and outcomes in these patients are excellent, with a four-year EFS of 92% and OS of 96% [44,45]. These tumors often spontaneously regress; therefore, a conservative approach was evaluated on a recent COG pilot study for perinatal neuroblastoma (ANBL00P2). The trial utilized observation only without biopsy or therapeutic intervention for patients less than six months of age at diagnosis with small adrenal masses (<16 mL in volume if solid and <65 mL in volume if ≥25% cystic). Of the subjects observed on this trial, 81% were spared surgical resection or chemotherapy with excellent event free and overall survival, 97.7% and 100%, respectively [45].

### 3.4. Stage MS Tumors

Studies have confirmed the favorable outcome in patients with MS disease with a five-year EFS rate for all infants of 86% and OS ranging 81–92%. Many patients in these trials were treated with supportive care alone [46]. The majority of infants with an MS pattern of disease have tumors with favorable biologic features, and many will experience spontaneous tumor regression without systemic therapy. Observation is the preferred strategy for clinically well patients with stage MS disease. There are subsets of patients with MS neuroblastoma who do not fare as well and experience recurrent disease as seen in patients 12–18 months of age with unfavorable tumor biology [47]. Others are at risk for significant morbidity and mortality particularly patients with large tumors and those with extensive liver involvement who develop respiratory compromise, abdominal compartment syndrome, venous obstruction, and liver dysfunction. In such patients, rapid initiation of chemotherapy to decrease tumor burden and alleviate symptoms is required, often without biopsy in very ill infants [46,48]. Outcome data from patients with INSS Stage 4S disease treated on A3961 and P9641 similarly suggest early initiation of treatment may be beneficial. The five-year OS rate for patients who were observed was 84.3%, but reached >97% for symptomatic patients who received treatment [37,38].

## 4. Relapsed Therapy

The approach to salvage therapy in patients with relapsed or refractory non-high-risk disease is not standardized and depends greatly on initial therapy and site of recurrence (localized or metastatic). For those patients who were observed or minimally treated up front it may be appropriate to consider surgical resection or begin/resume standard intermediate-risk therapy. For others, treatment has include second line chemotherapy regimen, combination chemotherapy, surgery, and radiation and even phase 1 or 2 clinical trials [49,50]. Irinotecan and temozolomide has demonstrated activity in patients with relapsed neuroblastoma particularly in combination with the anti-GD2 antibody dinutuximab, a regimen frequently used in patients with relapsed high-risk disease [51,52]. Cyclophosphamide and topotecan has also been shown to be an effective regimen in patients with relapsed or refractory neuroblastoma patients, and so is often utilized in these cases [53,54]. A secondary aim on ANBL0531 was to incorporate this chemotherapy combination as standard retrieval therapy to better describe outcomes following this approach in relapsed or refractory intermediate-risk patients.

## 5. Therapy—Future Directions

Classification of non-high-risk patients according to stage, age, histologic, and genomic characteristics allows for identification of those patient cohorts with excellent or only intermediate survival. Clinical and biologic characteristics present at diagnosis identify cohorts of non-high-risk neuroblastoma patients with survivals nearing 100% and in whom exposure to chemotherapy or surgery may be unnecessary. Conversely, there remain patients with non-high-risk neuroblastoma who die of their disease and in whom earlier initiation of treatment or modification of current therapy may be beneficial.

An ongoing non-high-risk neuroblastoma trial (ANBL1232) expands upon the perinatal experience extending the age cutoff to 12 months, increasing the upper limit of tumor size to 5 cm maximum diameter, and allowing for patients with non-adrenal primary tumors to be observed. Asymptomatic patients <18 months of age with localized tumors that have image defined risk factors (INRGSS L2 tumor) are being observed after biopsy only if the tumors are found to have favorable histology and favorable genomic features (absence of *MYCN* amplification and absence of segmental chromosomal aberrations). Allowing for spontaneous regression of tumors with biologically favorable features such that patients will not require neo-adjuvant chemotherapy or attempted surgical resection avoids all inherent risks of either intervention.

Patients with stage MS disease are likely to undergo spontaneous tumor regression and have an excellent outcome with observation only. Of concern are those patients with a large tumor or extensive liver involvement who are symptomatic and at risk for organ compromise. Chemotherapy or in rare cases radiation may be necessary to decrease tumor burden and alleviate symptoms. A scoring system to measure signs and symptoms of clinical deterioration has only been evaluated retrospectively, but was predictive of patient’s clinical course. The COG study ANBL1232 will be the first to incorporate a symptom-based scoring system in the MS patient cohort to be evaluated prospectively, validated, and then incorporated in future studies. This study will determine if intervening with immediate therapy or prior to the development of Grade 3 toxicity in the very young patients with hepatomegaly will improve outcome.

## Figures and Tables

**Table 1:** International Neuroblastoma Risk Group Staging System (INRGSS) [5,6].

INRG Stage	Description
**L1**	Localized tumor not involving vital structures as defined by absence of an image-defined risk factor and confined to one body compartment.
**L2**	Locoregional tumor with presence of one or more image-defined risk factors.
**M**	Distant metastatic disease (except MS).
**MS**	Metastatic disease in children younger than 18 months with metastases confined to skin, liver, and/or bone marrow (bone marrow involvement should be limited to <10% of total nucleated cells on smears or biopsy). Primary tumor may be L1 or L2 as defined above.

**Table 2:** Children’s Oncology Group (COG) neuroblastoma risk assignment table [34].

INSS Stage *	Age	*MYCN*	DNA Index	INPC	Other	Risk Classification
1	Any	Any	Any	Any		Low
2a/2b	Any	Not amplified	Any	Any	Resection ≥50%, asymptomatic	Low
2a/2b	Any	Not amplified	Any	Any	Resection ≥50%, symptomatic	Intermediate
2a/2b	Any	Not amplified	Any	Any	Resection <50%	Intermediate
2a/2b	Any	Not amplified	Any	Any	Biopsy only	Intermediate
2a/2b	Any	Amplified	Any	Any	Any degree of resection	High
3	<547 days	Not amplified	Any	Any		Intermediate
3	>547 days	Not amplified	Any	Favorable		Intermediate
3	Any	Amplified	Any	Any		High
3	>547 days	Not amplified	Any	Unfavorable		High
4	<365 d	Amplified	Any	Any		High
4	<365 d	Not amplified	Any	Any		Intermediate
4	365–<547 days	Amplified	Any	Any		High
4	365–<547 days	Any	DI = 1	Any		High
4	365–<547 days	Any	Any	Unfavorable		High
4	365–<547 days	Not amplified	DI > 1	Favorable		Intermediate
4	>547 days	Any	Any	Any		High
4S	<365 days	Not amplified	DI > 1	Favorable	Asymptomatic	Low
4S	<365 days	Not amplified	DI = 1	Any	Asymptomatic or symptomatic	Intermediate
4S	<365 days	Missing	Missing	Missing	Too sick for biopsy	Intermediate
4S	<365 days	Not amplified	Any	Any	Symptomatic	Intermediate
4S	<365 days	Not amplified	Any	Unfavorable	Asymptomatic or symptomatic	Intermediate
4S	<365 days	Amplified	Any	Any	Asymptomatic or symptomatic	High

* Modified schema under development replacing INSS with INRGSS. Reprinted with permission. © 2018 American Society of Clinical Oncology. All rights reserved. Naranjo, A. et al.: J Clin Oncol Clinical Cancer Informatics, Vol. (2), 2018: page 14.

**Table 3:** INRG neuroblastoma risk assignment table [5].

INRG Stage	Age (Months)	Histology	Tumor Differentiation	*MYCN* Status	11q Aberration	Ploidy	Risk Classification
L1/L2	Any	Ganglioneuroma (GN) maturing Ganglioneuroblastoma (GNB) intermixed	Any	Any	Any	Any	Very low
L1	Any	Any except GN maturing or GNB intermixed	Any	Non-amplified	Any	Any	Very low
L1	Any	Any except GN maturing or GNB intermixed	Any	Amplified	Any	Any	High
L2	<18	Any except GN maturing or GNB intermixed	Any	Non-amplified	No	Any	Low
L2	<18	Any except GN maturing or GNB intermixed	Any	Non-amplified	Yes	Any	Intermediate
L2	≥18	GNB nodular, neuroblastoma	Differentiating	Non-amplified	No	Any	Low
L2	≥18	GNB nodular, neuroblastoma	Differentiating	Non-amplified	Yes	Any	Intermediate
L2	≥18	GNB nodular, neuroblastoma	Poorly differentiated, undifferentiated	Non-amplified	Any	Any	Intermediate
L2	≥18	GNB nodular, neuroblastoma	Any	Amplified	Any	Any	High
M	<18	Any	Any	Non-amplified	Any	Hyperdiploid	Low
M	<12	Any	Any	Non-amplified	Any	Diploid	Intermediate
M	12 to <18	Any	Any	Non-amplified	Any	Diploid	Intermediate
M	<18	Any	Any	Amplified	Any	Any	High
M	≥18	Any	Any	Any	Any	Any	High
MS	<18	Any	Any	Non-amplified	No	Any	Very low
MS	<18	Any	Any	Non-amplified	Yes	Any	High
MS	<18	Any	Any	Amplified	Any	Any	High

Reprinted with permission. © 2009 American Society of Clinical Oncology. All rights reserved. Cohn, S.L. et al.: J Clin Oncol, Vol. (27:2), 2009: page 295.

**Table 4:** Comparison of standard upfront treatment for patients with neuroblastoma.

Risk Classification	Standard Therapy
Low Risk	Observation
	Surgical resection
Intermediate Risk	Chemotherapy
	Surgical resection
High Risk	Chemotherapy
	Surgical resection
	Myeloablative chemotherapy with autologous stem cell transplant
	External beam radiation therapy
	Immunotherapy with differentiating agent

## References

[1] Howlader N., Noone A.M., Krapcho M., Neyman N., Aminou R., Waldron W., Altekruse S.F., Kosary C.L., Ruhl J., Tatalovich Z. SEER Cancer Statistics Review, 1975–2009 (Vintage 2009 Populations).

[2] Cotterill S.J., Pearson A.D., Pritchard J., Foot A.B., Roald B., Kohler J.A. (2000). Clinical prognostic factors in 1277 patients with neuroblastoma: Results of The European Neuroblastoma Study Group ‘Survey’ 1982–1992. Eur. J. Cancer.

[3] London W.B., Boni L., Simon T., Berthold F., Twist C., Schmidt M.L., Castleberry R.P., Matthay K.K., Cohn S.L., De Bernardi B. (2005). The role of age in neuroblastoma risk stratification: The German, Italian, and children’s oncology group perspectives. Cancer Lett..

[4] London W.B., Castleberry R.P., Matthay K.K., Look A.T., Seeger R.C., Shimada H., Thorner P., Brodeur G., Maris J.M., Reynolds C.P. (2005). Evidence for an age cutoff greater than 365 days for neuroblastoma risk group stratification in the Children’s Oncology Group. J. Clin. Oncol..

[5] Cohn S.L., Pearson A.D., London W.B., Monclair T., Ambros P.F., Brodeur G.M., Faldum A., Hero B., Iehara T., Machin D. (2009). INRG Task Force. The International Neuroblastoma Risk Group (INRG) classification system: An INRG Task Force report. J. Clin. Oncol..

[6] Monclair T., Brodeur G.M., Ambros P.F., Brisse H.J., Cecchetto G., Holmes K., Kaneko M., London W.B., Matthay K.K., Nuchtern J.G. (2009). The International Neuroblastoma Risk Group (INRG) staging system: An INRG Task Force report. J. Clin. Oncol..

[7] Brodeur G.M., Seeger R.C., Barrett A., Castleberry R.P., D’Angio G., De Bernardi B., Evans A.E., Favrot M., Freeman A.I., Haase G. (1988). International criteria for diagnosis, staging, and response to treatment in patients with neuroblastoma. J. Clin. Oncol..

[8] Shimada H., Ambros I.M., Dehner L.P., Hata J., Joshi V.V., Roald B., Stram D.O., Gerbing R.B., Lukens J.N., Matthay K.K. (1999). The International Neuroblastoma Pathology Classification (the Shimada system). Cancer.

[9] Brodeur G.M., Seeger R.C., Schwab M., Varmus H.E., Bishop J.M. (1984). Amplification of N-*myc* in untreated human neuroblastomas correlates with advanced disease stage. Science.

[10] Pession A., De Bernardi B., Perri P., Mazzocco K., Rondelli R., Nigro M., Iolascon A., Forni M., Basso G., Conte M. (1994). The prognostic effect of amplification of the *MYCN* oncogene in neuroblastoma. The preliminary results of the Italian Cooperative Group for Neuroblastoma (GCINB). Pediatr. Med. Chir..

[11] Schwab M. (1993). Amplification of N-*myc* as a prognostic marker for patients with neuroblastoma. Semin. Cancer Biol..

[12] Campbell K., Gastier-Foster J.M., Mann M., Naranjo A.H., Van Ryn C., Bagatell R., Matthay K.K., London W.B., Irwin M.S., Shimada H. (2017). Association of *MYCN* copy number with clinical features, tumor biology, and outcomes in neuroblastoma: A report from the Children’s Oncology Group. Cancer.

[13] Eckschlager T., Pilat D., Kodet R., Dahbiova R., Stankova J. (1996). DNA ploidy in neuroblastoma. Neoplasma.

[14] Look A.T., Hayes F.A., Shuster J.J., Douglass E.C., Castleberry R.P., Bowman L.C., Smith E.I., Brodeur G.M., Jasinska J., Hrusak O. (1991). Clinical relevance of tumor cell ploidy and N-*myc* gene amplification in childhood neuroblastoma: A Pediatric Oncology Group study. J. Clin. Oncol..

[15] Attiyeh E.F., London W.B., Mosse Y.P., Wang Q., Winter C., Khazi D., McGrady P.W., Seeger R.C., Look A.T., Shimada H. (2005). Children’s Oncology Group. Chromosome 1p and 11q deletions and outcome in neuroblastoma. N. Engl. J. Med..

[16] Caron H., van Sluis P., de Kraker J., Bokkerink J., Egeler M., Laureys G., Slater R., Westerveld A., Voute P.A., Versteeg R. (1996). Allelic loss of chromosome 1p as a predictor of unfavorable outcome in patients with neuroblastoma. N. Engl. J. Med..

[17] Fong C.T., Dracopoli N.C., White P.S., Merrill P.T., Griffith R.C., Housman D.E., Brodeur G.M. (1989). Loss of heterozygosity for the short arm of chromosome 1 in human neuroblastomas: Correlation with N-*myc* amplification. Proc. Natl. Acad. Sci. USA.

[18] Guo C., White P.S., Weiss M.J., Hogarty M.D., Thompson P.M., Stram D.O., Gerbing R., Matthay K.K., Seeger R.C., Brodeur G.M. (1999). Allelic deletion at 11q23 is common in *MYCN* single copy neuroblastomas. Oncogene.

[19] Maris J.M., Guo C., White P.S., Hogarty M.D., Thompson P.M., Stram D.O., Gerbing R., Matthay K.K., Seeger R.C., Brodeur G.M. (2001). Allelic deletion at chromosome bands 11q14-23 is common in neuroblastoma. Med. Pediatr. Oncol..

[20] Maris J.M., Weiss M.J., Guo C., Gerbing R.B., Stram D.O., White P.S., Hogarty M.D., Sulman E.P., Thompson P.M., Lukens J.N. (2000). Loss of heterozygosity at 1p36 independently predicts for disease progression but not decreased overall survival probability in neuroblastoma patients: A Children’s Cancer Group study. J. Clin. Oncol..

[21] Maris J.M., White P.S., Beltinger C.P., Sulman E.P., Castleberry R.P., Shuster J.J., Look A.T., Brodeur G.M. (1995). Significance of chromosome 1p loss of heterozygosity in neuroblastoma. Cancer Res..

[22] Schleiermacher G., Michon J., Huon I., d’Enghien C.D., Klijanienko J., Brisse H., Ribeiro A., Mosseri V., Rubie H., Munzer C. (2007). Chromosomal CGH identifies patients with a higher risk of relapse in neuroblastoma without *MYCN* amplification. Br. J. Cancer.

[23] Schleiermacher G., Michon J., Ribeiro A., Pierron G., Mosseri V., Rubie H., Munzer C., Benard J., Auger N., Combaret V. (2011). Segmental chromosomal alterations lead to a higher risk of relapse in infants with *MYCN*-non-amplified localised unresectable/disseminated neuroblastoma (a SIOPEN collaborative study). Br. J. Cancer.

[24] Pinto N., Mayfield J.R., Raca G., Applebaum M.A., Chlenski A., Sukhanova M., Bagatell R., Irwin M.S., Little A., Rawwas J. (2016). Segmental Chromosomal Aberrations in Localized Neuroblastoma Can be Detected in Formalin-Fixed Paraffin-Embedded Tissue Samples and Are Associated With Recurrence. Pediatr. Blood Cancer.

[25] Defferrari R., Mazzocco K., Ambros I.M., Ambros P.F., Bedwell C., Beiske K., Benard J., Berbegall A.P., Bown N., Combaret V. (2015). Influence of segmental chromosome abnormalities on survival in children over the age of 12 months with unresectable localised peripheral neuroblastic tumours without *MYCN* amplification. Br. J. Cancer.

[26] Tanaka K., Arif M., Eguchi M., Guo S.X., Hayashi Y., Asaoku H., Kyo T., Dohy H., Kamada N. (1991). A significant association of Ha-ras p21 in neuroblastoma cells with patient prognosis. A retrospective study of 103 cases. Cancer.

[27] de Souza D.R., Sanabani S.S., de Souza A.C., Filho Odone V., Epelman S., Bendit I. (2011). Prognostic impact of *MYCN*, *DDX1*, *TrkA*, and *TrkC* gene transcripts expression in neuroblastoma. Pediatr. Blood Cancer.

[28] Kogner P., Barbany G., Dominici C., Castello M.A., Raschella G., Persson H. (1993). Coexpression of messenger RNA for TRK protooncogene and low affinity nerve growth factor receptor in neuroblastoma with favorable prognosis. Cancer Res..

[29] Gattolliat C.H., Le Teuff G., Combaret V., Mussard E., Valteau-Couanet D., Busson P., Benard J., Douc-Rasy S. (2014). Expression of two parental imprinted miRNAs improves the risk stratification of neuroblastoma patients. Cancer Med..

[30] Nguyen le B., Diskin S.J., Capasso M., Wang K., Diamond M.A., Glessner J., Kim C., Attiyeh E.F., Mosse Y.P., Cole K. (2011). Phenotype restricted genome-wide association study using a gene-centric approach identifies three low-risk neuroblastoma susceptibility Loci. PLoS Genet..

[31] Oberthuer A., Juraeva D., Hero B., Volland R., Sterz C., Schmidt R., Faldum A., Kahlert Y., Engesser A., Asgharzadeh S. (2015). Revised risk estimation and treatment stratification of low- and intermediate-risk neuroblastoma patients by integrating clinical and molecular prognostic markers. Clin. Cancer Res..

[32] Warnat P., Oberthuer A., Fischer M., Westermann F., Eils R., Brors B. (2007). Cross-study analysis of gene expression data for intermediate neuroblastoma identifies two biological subtypes. BMC Cancer.

[33] Vermeulen J., De Preter K., Naranjo A., Vercruysse L., Van Roy N., Hellemans J., Swerts K., Bravo S., Scaruffi P., Tonini G.P. (2009). Predicting outcomes for children with neuroblastoma using a multigene-expression signature: A retrospective SIOPEN/COG/GPOH study. Lancet Oncol..

[34] Naranjo A., Irwin M.S., Hogarty M.D., Cohn S.L., Park J.R., London W.B. (2018). Statistical Framework in Support of a Revised Children’s Oncology Group Neuroblastoma Risk Classification System. JCO Clin. Cancer Inform..

[35] Weinstein J.L., Katzenstein H.M., Cohn S.L. (2003). Advances in the diagnosis and treatment of neuroblastoma. Oncologist.

[36] Park J.R., Eggert A., Caron H. (2010). Neuroblastoma: Biology, prognosis, and treatment. Hematol. Oncol. Clin. N. Am..

[37] Strother D.R., London W.B., Schmidt M.L., Brodeur G.M., Shimada H., Thorner P., Collins M.H., Tagge E., Adkins S., Reynolds C.P. (2012). Outcome after surgery alone or with restricted use of chemotherapy for patients with low-risk neuroblastoma: Results of Children’s Oncology Group study P9641. J. Clin. Oncol..

[38] Baker D.L., Schmidt M.L., Cohn S.L., Maris J.M., London W.B., Buxton A., Stram D., Castleberry R.P., Shimada H., Sandler A. (2010). Outcome after reduced chemotherapy for intermediate-risk neuroblastoma. N. Engl. J. Med..

[39] De Bernardi B., Mosseri V., Rubie H., Castel V., Foot A., Ladenstein R., Laureys G., Beck-Popovic M., de Lacerda A.F., Pearson A.D. (2008). Treatment of localised resectable neuroblastoma. Results of the LNESG1 study by the SIOP Europe Neuroblastoma Group. Br. J. Cancer.

[40] Matthay K.K., Perez C., Seeger R.C., Brodeur G.M., Shimada H., Atkinson J.B., Black C.T., Gerbing R., Haase G.M., Stram D.O. (1998). Successful treatment of stage III neuroblastoma based on prospective biologic staging: A Children’s Cancer Group study. J. Clin. Oncol..

[41] Kohler J.A., Rubie H., Castel V., Beiske K., Holmes K., Gambini C., Casale F., Munzer C., Erminio G., Parodi S. (2013). Treatment of children over the age of one year with unresectable localised neuroblastoma without *MYCN* amplification: Results of the SIOPEN study. Eur. J. Cancer.

[42] Hero B., Simon T., Spitz R., Ernestus K., Gnekow A.K., Scheel-Walter H.G., Schwabe D., Schilling F.H., Benz-Bohm G., Berthold F. (2008). Localized infant neuroblastomas often show spontaneous regression: Results of the prospective trials NB95-S and NB97. J. Clin. Oncol..

[43] Twist C., London W.B., Naranjo A., Schmidt M.L., Adkins E.S., Mattei P., Cretella S., Cohn S.L., Park J.R., Maris J.M. Maintaining outstanding outcomes using response- and biology-based therapy for intermediate-risk neuroblastoma: A report from the Children’s Oncology Group study ANBL0531. Proceedings of the American Society of Clinical Oncology, Annual Meeting.

[44] Acharya S., Jayabose S., Kogan S.J., Tugal O., Beneck D., Leslie D., Slim M. (1993). Prenatally diagnosed neuroblastoma. Cancer.

[45] Nuchtern J.G., London W.B., Barnewolt C.E., Naranjo A., McGrady P.W., Geiger J.D., Diller L., Schmidt M.L., Maris J.M., Cohn S.L. (2012). A prospective study of expectant observation as primary therapy for neuroblastoma in young infants: A Children’s Oncology Group study. Ann. Surg..

[46] Nickerson H.J., Matthay K.K., Seeger R.C., Brodeur G.M., Shimada H., Perez C., Atkinson J.B., Selch M., Gerbing R.B., Stram D.O. (2000). Favorable biology and outcome of stage IV-S neuroblastoma with supportive care or minimal therapy: A Children’s Cancer Group study. J. Clin. Oncol..

[47] Taggart D.R., London W.B., Schmidt M.L., DuBois S.G., Monclair T.F., Nakagawara A., De Bernardi B., Ambros P.F., Pearson A.D., Cohn S.L. (2011). Prognostic value of the stage 4S metastatic pattern and tumor biology in patients with metastatic neuroblastoma diagnosed between birth and 18 months of age. J. Clin. Oncol..

[48] Twist C.J., Naranjo A., Schmidt M.L., Tenney S.C., Cohn S.L., Meany H.J., Mattei P., Adkins E.S., Shimada H., London W.B. (2018). Defining Risk Factors for Chemotherapeutic Intervention in Infants with Stage 4S Neuroblastoma: A Report from Children’s Oncology Group Study ANBL0531. J. Clin. Oncol..

[49] Basta N.O., Halliday G.C., Makin G., Birch J., Feltbower R., Bown N., Elliott M., Moreno L., Barone G., Pearson A.D. (2016). Factors associated with recurrence and survival length following relapse in patients with neuroblastoma. Br. J. Cancer.

[50] London W.B., Bagatell R., Weigel B.J., Fox E., Guo D., Van Ryn C., Naranjo A., Park J.R. (2017). Historical time to disease progression and progression-free survival in patients with recurrent/refractory neuroblastoma treated in the modern era on Children’s Oncology Group early-phase trials. Cancer.

[51] Bagatell R., London W.B., Wagner L.M., Voss S.D., Stewart C.F., Maris J.M., Kretschmar C., Cohn S.L. (2011). Phase II study of irinotecan and temozolomide in children with relapsed or refractory neuroblastoma: A Children’s Oncology Group study. J. Clin. Oncol..

[52] Mody R., Naranjo A., Van Ryn C., Yu A.L., London W.B., Shulkin B.L., Parisi M.T., Servaes S.E., Diccianni M.B., Sondel P.M. (2017). Irinotecan-temozolomide with temsirolimus or dinutuximab in children with refractory or relapsed neuroblastoma (COG ANBL1221): An open-label, randomised, phase 2 trial. Lancet Oncol..

[53] Ashraf K., Shaikh F., Gibson P., Baruchel S., Irwin M.S. (2013). Treatment with topotecan plus cyclophosphamide in children with first relapse of neuroblastoma. Pediatr. Blood Cancer.

[54] London W.B., Frantz C.N., Campbell L.A., Seeger R.C., Brumback B.A., Cohn S.L., Matthay K.K., Castleberry R.P., Diller L. (2010). Phase II randomized comparison of topotecan plus cyclophosphamide versus topotecan alone in children with recurrent or refractory neuroblastoma: A Children’s Oncology Group study. J. Clin. Oncol..

